# Whose rights deserve protection? Framing analysis of responses to the 2016 Committee of Advertising Practice consultation on the non-broadcast advertising of foods and soft drinks to children

**DOI:** 10.1016/j.foodpol.2021.102139

**Published:** 2021-10

**Authors:** Lauren Carters-White, Stephanie Chambers, Kathryn Skivington, Shona Hilton

**Affiliations:** a MRC/CSO Social and Public Health Science Unit, University of Glasgow, Berkeley Square, 99 Berkeley Street, Glasgow G3 7HR, United Kingdom; b SPECTRUM Consortium, Usher Institute of Population Health Sciences and Informatics, Doorway 1, Old Medical School, Teviot Place, University of Edinburgh, Edinburgh EH8 9AG, United Kingdom; c School of Social and Political Sciences, University of Glasgow, United Kingdom

**Keywords:** Framing, Childhood obesity, Marketing, Values, Regulation

## Abstract

Exposure to advertising of food and beverages high in fat sugar and salt (HFSS) is considered a factor in the development of childhood obesity. This paper uses framing analysis to examine the strategic discursive practices employed by non-industry and industry responders to the Committee of Advertising Practice’s consultation responses (n = 86) on UK regulation of non-broadcast advertising of foods and soft drinks to children. Our analysis demonstrates non-industry and industry responders engaged in a moral framing battle centred on whose rights were deemed as being of greatest importance to protect: children or industry. Both industry and non-industry responders acknowledged that childhood obesity and non-broadcast advertising were complex issues but diverged on how they morally framed their arguments. Non-industry responders employed a moral framework that aligned with the values represented in social justice approaches to public health policy, where children were identified as vulnerable, in need of protection from harmful HFSS product advertising and childhood obesity was a societal problem to solve. In contrast, industry responders emphasised industry rights, portraying themselves as a responsible industry that is victim to perceived disproportionate policymaking, and values more closely aligned with a market justice approach to public health policy. Our analysis provides detailed insights into the framing strategies used in the policy debate surrounding the non-broadcast advertising of HFSS foods to children. This has relevance as to how advocacy organisations can develop counter-framing to industry frames which seek to limit effective regulation.

## Highlights

• Food advertising regulation a contentious policy issue.
• Framing strategies used by policy actors important to capture for policy analysis.
• Policy actors use different moral frameworks to support or oppose policies.
• Tension between children’s rights and industry rights moral frameworks identified.

## 1 Introduction

Exposure to the advertising of food and beverages high in fat, sugar and salt (HFSS) is considered a contributory factor in the development of overweight and obesity in children (Hastings et al., 2003, Swinburn et al., 2011, Smith et al., 2019). There is increasing evidence as to the impact such advertising has on children’s dietary preferences, particularly that which occurs in the non-broadcast environment (Baldwin et al., 2018, Coates et al., 2019b, Coates et al., 2019a, Critchlow et al., 2019, Smith et al., 2019). Non-broadcast advertising is that which is not disseminated via television or radio, and most often refers to advertising that occurs in the online environment, but can also include other advertising mediums such as billboards or posters at transports stops. In the United Kingdom (UK), and globally, this has led to increased calls to strengthen the regulations surrounding the non-broadcast advertising of HFSS products to children as part of a suite of policy interventions to reduce childhood obesity rates (Tedstone et al., 2015, House of Commons Health Social Care Committee, 2018).

In response to concerns regarding childhood obesity, the UK Government announced new restrictions and future consultations for non-broadcast advertising of HFSS products in the summer of 2020 (UK Government, 2020a). This announcement follows a series of recent government inquiries and consultations examining childhood obesity in the UK, commencing in 2015 with the Childhood Obesity Inquiry by the UK Government’s Health & Social Care Committee (House of Commons Health & Social Care Committee, 2016). Despite regular reference to the role non-broadcast advertising of HFSS products plays in contributing to an obesogenic environment, there has, as of yet, been minimal policy action to address these concerns. However, the UK Government has recently announced a specific ban on online advertising of HFSS products, due to be implemented at the end of 2022 (UK Government, 2021).

In the UK, the regulation of non-broadcast advertising of HFSS products is undertaken by the Committee of Advertising Practice (CAP) and the Advertising Standards Agency (ASA). This system is a self-regulatory one, with the ASA and CAP stating it is effective *“because it is powered and driven by a sense of corporate social responsibility amongst the advertising industry”* (ASA, 2020). The CAP is responsible for writing and maintaining the regulatory Code, and the ASA is responsible for administering and enforcing the Code. Despite CAP’s and ASA’s assertion as to the robustness of self-regulation, there has been increasing criticism of the effectiveness of self-regulatory systems (Hawkes, 2005, Chambers et al., 2015, Lacy-Nichols et al., 2020). Additionally, there are criticisms of industry self-regulation as a corporate social responsibility (CSR) initiative, allowing industry to exercise rule-setting power that protects their industry from the perceived risk of disproportionate regulation (Fuchs and Lederer, 2007), as well as improving their public and policy reputation (Dorfman et al., 2012). As seen above, CAP and ASA openly describe their self-regulation as CSR. Such CSR initiatives can perform as a public relations activity, allowing industry to portray ‘innocence by association’ (Dorfman et al., 2012), and they are often enacted in response to when corporations perceive a threat to their profit-generating goals (Siegel and Vitaliano, 2007). By undertaking the regulation of non-broadcast advertising, industry are simultaneously portraying themselves as a ‘responsible’ industry through the protection of children from harmful advertising, as well as tempering the risk of regulation negatively impacting on their profit-generating goals.

**Fig. 1:**
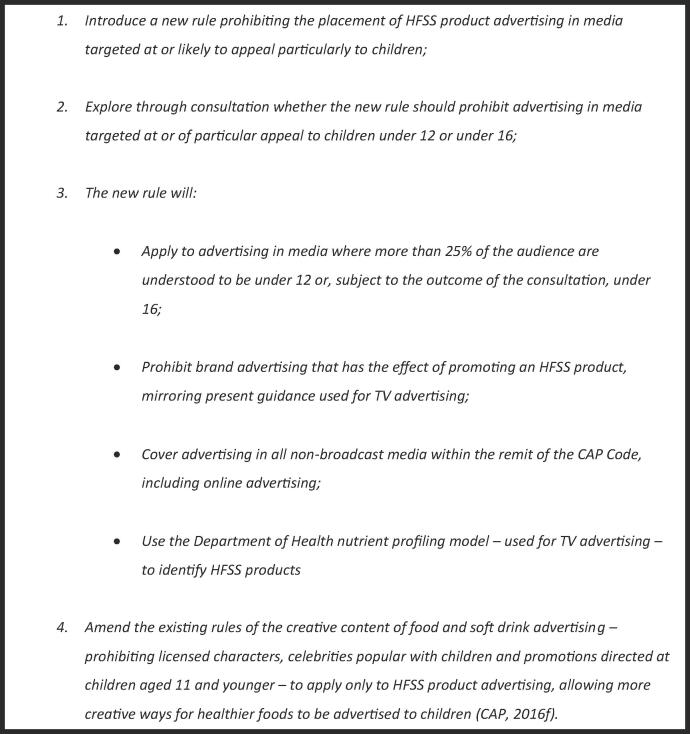
Proposed policies in CAP Consultation: Food and Soft Drink Advertising to Children (CAP, 2016f).

In 2016, in response to the UK 2015 Childhood Obesity Inquiry (House of Commons Health & Social Care Committee, 2016), the CAP launched their public consultation titled *CAP Consultation: Food and Soft Drink Advertising to Children* (CAP, 2015) from the 13th May 2016 to the 22nd July 2016. This consultation was designed to address concerns regarding the effectiveness of the self-regulatory system raised during the 2015 Childhood Obesity Inquiry, and this arguably represents the CAP and ASA attempting to enact a CSR initiative in response to the perceived threat of statutory regulation (as was recommended during the inquiry) (Siegel and Vitaliano, 2007). The 2016 CAP consultation was also the first meaningful consultation on advertising of HFSS product regulation in the UK since the 2007 Ofcom consultation on broadcast advertising (Razavi et al., 2019). The 2016 CAP consultation sought to strengthen the regulations surrounding non-broadcast advertising, as well as bring these regulations in line with broadcast (television and radio) regulations. Fig. 1 describes the proposals CAP presented for consultation.

A range of non-industry (advocacy, advisory, government body and academics) and industry (food and beverage industry, advertising industry, retailers and media) stakeholders responded to the consultation. The CAP consultation resulted in the strengthening of non-broadcast advertising of HFSS products as announced in December 2016, which formally came into effect on 1st July 2017 (CAP, 2016g). However, despite the changes in advertising regulation as a result of this consultation, and as far as the researchers are aware, there has been no critical examination of the responses to this consultation. This is despite previous research highlighting that consultations are a key site in which policy actors enter to influence policy development (Jenkin et al., 2011, Hawkins and Holden, 2013, Scott et al., 2017, Lauber et al., 2020), and examining consultations provides an important opportunity to understand how organisational interests may guide policy development. Previous consultation framing analyses examining food policy globally have shown that industry utilise these forums to ‘water down’ policy proposals (Razavi et al., 2019), as well as to promote industry as necessary and legitimate policy partners (Lauber et al., 2020). This is particularly important in the case of non-broadcast advertising of HFSS products, as its role in contributing to childhood overweight and obesity remains a politically contested issue (Jenkin et al., 2011, Nimegeer et al., 2019, Razavi et al., 2019, Russell et al., 2020). The industry and non-industry actors who engaged in the CAP consultation may have used varied arguments to influence the policy development, and examining these varied arguments can bring to the fore how actors structure and frame their responses to influence policy to either undermine and negate against self-regulation, or to protect the self-regulatory model (Beauchamp, 1976, Dorfman et al., 2005). However, as far as the researchers are aware, there has been no such close examination of the framing practices employed by policy actors in the UK in relation to the non-broadcast advertising of HFSS products, despite the increase in policy attention. As such, there is a gap in our understanding as to how multiple actors construct arguments and discursively justify supporting or opposing non-broadcast HFSS product advertising regulation and legitimise their position for doing so. Examining the responses to the CAP consultation provides a unique opportunity to conduct such an analysis.

Framing analysis, based on frame theory, is one approach to investigate how such contested problems are constructed (Entman, 1993), allowing for comparisons of how different stakeholders react to policy issues (Chong and Druckman, 2007, Russell et al., 2020). In the case of policymaking, understanding framing through a systematic analysis approach can therefore help to explain the success of some policy issues or solutions, and why others fail to be implemented or even acknowledged.

Previous research examining obesity policy debates has argued that these debates are often underpinned by two broad frames, termed as social justice and market justice, which provide a symbolic framework in which to think about and react to policy problems (Beauchamp, 1976, Lawrence, 2004, Dorfman et al., 2005, Coleman et al., 2011, Jenkin et al., 2011, Shugart, 2011, Hilton et al., 2012, Barry et al., 2013, Gollust et al., 2013, Jeong et al., 2014, Patchett et al., 2014, Ortiz et al., 2016, Russell et al., 2020). These symbolic frameworks provide the *“basic rules to classify and categorize problems of society as to whether they necessitate public and collective protection, or whether individual responsibility should prevail”* (Beauchamp, 1976, p4). Dorfman et al. (2005) posit that public health and UCIs engage in framing battles that are guided by these core morals, values and norms that align with these wider frames of social and market justice.

As described above, one approach for documenting and understanding such framing battles is framing analysis (Beauchamp, 1976, Entman, 1993, Lakoff, 1996, Russell et al., 2020). Framing analysis proposes that the way in which an issue is presented to an audience – ‘the frame’ – influences how that audience processes that information, and as such is an effective tool in exerting political influence and power (Entman, 1993). Entman (1993, p53) defines framing as:

*Framing essentially involves selection and salience. To frame is to select some aspects of perceived reality and make them more salient in a communicating text, in such a way as to promote a particular****problem definition****,****causal interpretation****,****moral evaluation****, and/or****treatment recommendation****for the item described.*

Entman’s (1993) definition of framing assumes an intentionality behind the deployment of a frame, and is particularly useful for understanding the communication strategies of various actors within policy debates. This includes political actors’ use of discourse within the policy process to influence policymaking in line with their interests (Coburn, 2006) using the four signature rhetorical devices of problem definition, causal interpretation, moral evaluation and treatment recommendations.

As such, this paper seeks to conduct a detailed framing analysis of the 2016 CAP consultation responses on the non-broadcast advertising of foods and soft drinks to children, to investigate the strategic discursive practices employed by non-industry and industry responders. It conducts a nuanced and detailed analysis of the frames and their constituent parts contained within these responses, from both industry and non-industry organisations, to gain an in-depth understanding of how these frames are constructed, in order to understand how responding to such a consultation forms a part of policy actors’ political strategies.

## 2 Methods

### 2.1 Data collection

The responses to the aforementioned CAP consultation were accessed and downloaded from the CAP website in December 2016 following their publication along with the outcome report of the consultation (CAP, 2016a, CAP, 2016b, CAP, 2016c, CAP, 2016d, CAP, 2016e). As this study did not involve primary data collection with human participants, ethical approval was not required.

### 2.2 Data analysis

To operationalise Entman’s (1993) definition and to ensure the systematic identification and recording of key characteristics of frames, this analysis employed and amended the framing matrix developed by Jenkin et al. (2011) (see Table 1). The study team decided that although the definition provided by Entman (1993) and the framework by Jenkin et al. (2011) are useful, they do not place enough emphasis on the role that the overarching moral values, as recommended by Dorfman et al., 2005, Lakoff, 1996, plays in structuring the frames deployed by actors. As such, employing framing theory, but following Dorfman et al., 2005, Lakoff, 1996 recommendation that morals and values provide the overall framework, this analysis began by considering the moral evaluation performed by the consultation responders, which guided their problem definition, causal interpretation and recommended policy solutions. This is the first such detailed analysis using this systematic framing matrix, examining the framing strategies of both industry and non-industry actors related to the regulation of non-broadcast advertising of HFSS products in the UK.

**Table 1:** Framing matrix modified from Jenkin et al. (2011, p1025).

Signature rhetorical devices	Key aspects	Prompts
Moral evaluation	Core values or principles	What values or principles are evident in the problem representation?
Problem definition	Overall description	How is issue described? What is the emphasis? Why is the issue a problem?
	Type of problem	What type of problem is it?
	Affected groups	Who is the issue a problem for?
Causal interpretation	Main cause	What is identified as the main cause? Is the cause environmental or individual? Who/what is to blame for the problem?
	Non-causes	What are dismissed or explicitly identified as non-causes?
Recommended policy solutions (treatment recommendation)	Policy prescriptions	What solutions are proposed/emphasised? What issues are included? Are the solutions targeted or universal? Who is responsible?
	Non-solutions	What issues are excluded? What solutions are opposed?
	Existing policy	What are the views on current policy?

The framing matrix (Table 1) allowed for a systematic approach to identify the frames employed by responders to the consultation. Column one contains the signature rhetorical devices identified in Entman’s (1993) definition of framing, however *treatment recommendation* was replaced with *recommended policy solutions* as this was deemed a more appropriate term for the study context. The signature rhetorical devices formed the basis for the initial broad coding of the consultation responses. The second and third columns, labelled *key aspects* and *prompts*, provided further information and guidance for the systematic coding of the responses, and these formed the basis for more detailed and granular coding. In addition, if evidence or statistics were used in the responses, these were also coded to the appropriate signature rhetorical device to broadly understand how different responders employed the evidence base, if at all. However, it must be noted that an analysis of the type of evidence was not possible due to the CAP removing responses’ reference lists prior to publication. The coding was an iterative process and required repeated applications of the framing matrix to the consultation responses. As highlighted by Jenkin et al., 2011, Van Gorp, 2010, this iterative process is consistent with the constant comparative method, whereby texts are repeatedly compared to other texts within the sample. This allows for the generation of frames that may not be initially apparent, and which are only generated through the comparison to other frames.

The coding generated an extensive range of codes, and not all have been included in this final paper. The most directly relevant codes to the research’s aim have been included below. For codes that spanned multiple categories in the framing matrix, these codes were reported under the category which the code was of most direct relevance to.

### 2.3 Notes on methods and terminology

In the context of this study, when referring to consultations, we are referring to open public consultations, whereby multiple stakeholders can contribute (e.g. public, civil society, industry, government bodies). As seen in previous consultation analyses examining industry responses to obesity policies (Scott et al., 2017), when referring to ‘industry’ as a whole, it is recognised that within this sector there are multiple actors or sub-sectors with varying objectives and political priorities. Within this analysis, those sub-sectors were: 1) food and beverage industry, 2) advertising industry, 3) retailers and 4) media. These sub-sectors are referred to together as ‘industry responders’, as much of their responses aligned. Where their responses were not aligned, the individual sectors are described. Similarly, advocacy, advisory, government body and academic organisations have been assimilated as non-industry responders, as many of their responses aligned.

## 3 Results

In total, 86 different responses were listed in the consultation document: civil society (n = 45), industry (n = 23), government body (n = 8), media (n = 8) and public (n = 2). Non-industry responders (civil society, government body, and public) predominantly consisted of advocacy and advisory organisations. Industry responders predominantly consisted of large companies, and included a small number of trade organisations.

Table 2 below depicts the summary of framing devices found through the analyses according to the four signature rhetorical devices.

**Table 2:** Summary of non-industry and industry framing matrix.

Signature rhetorical device	Key aspects	Non-industry (public health)	Industry
Moral evaluation	Core values or principles	Social justice, children’s right to health, protection of vulnerable children	Market justice, rights of industry, protection of industry, responsible industry
Problem definition	Overall description	A complex issue, epidemic, concern for society (high use of statistics)	A complex issue, concern for consumers (very limited use of statistics)
	Type of problem	Unbounded problem, health/financial burden, significant threat to public health	Bounded problem, omission of consequences of obesity, a societal problem/debate
	Affected groups	All children (0–17 years-of-age), future adults, parents, lower socioeconomic groups	Children under 12, parents (only F&B industry)
Causal interpretation	Main cause	Obesogenic environment, multi-setting advertising and marketing practices, online advertising particularly problematic, children’s vulnerability (all ages), reliance on evidence and specific examples	Individual behaviours, children’s changing media habits, children’s vulnerability (under 12 years), limited reliance on evidence
Recommended policy solutions	Policy prescriptions	Implementation of recommended policy proposals to protect children (with further strengthening), ban on advertising of HFSS products	Implementation of recommended policy solutions to support a responsible industry, restriction on advertising of HFSS products
	Non-solutions	Piecemeal approach to reduction of childhood obesity, weak policies (maintenance of loopholes)	Statutory/disproportionate regulation, restricting advertising to reduce childhood obesity
	Existing policy	Critical of self-regulation, sceptical of industry practices	Success of self-regulation, advertising industry leaders in effective policy

### 3.1 Moral evaluation

Moral evaluation refers to the core values or principles evident within, or underpinning, a frame (Entman, 1993, Jenkin et al., 2011), and Lakoff (1996) posits that it is these morals and values that contribute to the development of a successful or effective framing approach. Throughout the consultation, both industry and non-industry responders took a ‘rights-based’ approach to framing, but diverged on whose rights were important to protect. The ‘rights-based’ framing drew on concepts of fairness, responsibility, and vulnerability. Non-industry responders were more likely to employ framing which supported a social justice approach to resolving childhood overweight and obesity, whereas industry responders were more likely to employ framing which supported a market justice approach (Beauchamp, 1976).

Non-industry responders took a child-centred approach to their framing, focusing on wider government and societal roles in ensuring the best possible protection was afforded to this vulnerable population. To support such framing, they made frequent reference to children’s right to health as a moral justification for supporting the policy proposals, for example by drawing attention to specific legal frameworks such as the United Nations (UN) Convention on the Rights of the Child (The United Nations, 1990):

*Current alarming rates of childhood obesity breach rights to health,* e.g. *children’s rights to development and enjoyment of the highest attainable standards of health as articulated in the UN Convention on the Rights of the Child (UNCRC; UN, 1990).* (Academic responder) (CAP, 2016a)

These frameworks were also employed by advocacy, advisory and academic responders to infer that governments, and wider society, should play a larger role in protecting children’s rights.

In contrast, industry responders took an industry-centred approach to their framing, positioning themselves as ‘responsible’. Industry responders acknowledged the need to protect children, however balanced this with the need to protect industry:

*We are focused on our responsibility to our patrons…we recognise the role that our sector has in playing its part within a framework, aimed at reducing children’s exposure to the HFSS advertising. It is also important that brands rights to advertise is protected.* (Advertising industry responder) (CAP, 2016b)

In addition, industry responders positioned their organisations as victims of perceived disproportionate public health policy and over-regulation:

*… the new provisions must allow industry to continue to operate in a competitive environment by avoiding the imposition of disproportionate burdens.* (Food and drink industry responder) (CAP, 2016a)

As a response to this perceived risk of disproportionate regulation, industry responders used an industry-centred approach to advocate for proportionate self-regulation as the most effective means to balance industry rights with the protection of children.

### 3.2 Problem definition

Problem definition is when actors define the ‘boundaries’ of an issue, emphasising an overall description of the problem, the type of problem and affected groups (Entman, 1993, Coburn, 2006, Jenkin et al., 2011). Within this consultation, both industry and non-industry responders framed childhood obesity as an important and complex problem requiring a multi-organisational response. However, differences existed as to how industry and non-industry responders described the magnitude of the childhood obesity problem.

Non-industry responders employed epidemic framing that defined childhood obesity as causing drastic detrimental impacts across the life-course of children and throughout society, often employing statistics on prevalence to strengthen their point:

*We have an obesity epidemic – in 2014, 65% of adults aged 16 and over were overweight, including 28% who were obese.* (Government body responder) (CAP, 2016e)

They described childhood obesity as a concern for the whole of society, employing language imbued with a sense of urgency for public health to act. When discussing the type of problem, non-industry responders framed childhood obesity as a health and financial burden that had far-reaching consequences across children’s life course and the wider population, effectively positioning it as an unbounded whole-population problem:

*As a consequence of health harms, the economic burden of obesity is staggering.* (Advocacy organisation responder) (CAP, 2016b)

Additionally, non-industry responders employed language that added to their epidemic framing through using terms such as *‘threat’* and *‘devastating’*.

Non-industry responders said all children under the age of 18 should be included in the updated CAP Code, as well as specifically highlighting those from lower socioeconomic groups as particularly vulnerable. In addition, non-industry responders identified parents and future adults as an affected group:

*In addition, misleading health or nutrition claims online and on packaging…skew the information parents are relying on when making purchasing decisions.* (Advocacy organisation responder) (CAP, 2016c)

In contrast, industry responders, despite acknowledging that childhood obesity was a complex issue, omitted this epidemic framing in their responses and tended to downplay the issue of obesity to one *‘of concern’* for their consumers. Industry were arguably attempting to minimise the public health need to reduce childhood overweight and obesity rates:

*Although advertising of food and soft drinks to children is only one small part of a very complex problem, it is a cause for concern amongst our customers.* (Food and drink industry responder) (CAP, 2016a)

There was a notable omission of the consequences of childhood overweight and obesity from industry responders and this may indicate industry attempts to frame childhood obesity as a bounded problem. In addition, industry responders employed less emotive language compared to non-industry responders’ ‘epidemic’ framing. Some responses noted childhood obesity as a *“serious problem”*, some as an *“obesity debate”* and others as an *“obesity problem”*. Furthermore, industry were specific in their assertion that it was children under the age of 12 who were the vulnerable population, as they did not possess enough media literacy over advertising content:

*We believe that it is right to protect children under 12 years old from all advertising HFSS as the scientific evidence suggests that they cannot identify and understand advertising’s persuasive intent before this age.* (Food and drink industry responder) (CAP, 2016c)

Industry deliberately separated children under 12-years-of-age from children under 16-years-of-age. By doing so, they narrowed attention to only these age groups, potentially to restrict how widely the policy proposals would be applied.

### 3.3 Causal interpretation

Causal interpretation is the process of allocating causes for a defined problem (Entman, 1993). Non-industry responders identified the non-broadcast advertising of HFSS products, as well as wider marketing practices, as contributing to poor dietary health for children across all ages. Non-industry responders dedicated considerable focus to explaining the effect non-broadcast advertising of HFSS products has on children’s dietary preferences, strengthening their argument by referring to research or evidence. Additionally, non-industry responders repeatedly referred to the obesogenic environment as the overarching causal factor in childhood overweight and obesity:

*Research shows that marketing greatly influences the food children choose to eat. It also increases the amount of food they eat. Marketing is a pivotal factor in the obesogenic environment, and tackling children’s obesity cannot be done effectively without restrictions on marketing to children.* (Advocacy organisation responder) (CAP, 2016d)

In contrast, industry responders were extremely specific in their causal interpretation and tended to highlight individual behaviours, such as knowledge deficits in parents and children, lack of physical activity and children’s changing media habits. Although industry responders acknowledged the potential impact non-broadcast advertising may have on children’s dietary preferences, they were explicit in their assertion that the evidence base demonstrated it was children under 12 years-of-age who were vulnerable to such effects:

*We believe our science-based approach to understanding child appeal should be applied industry-wide in order to ensure that children under 12 years old who are not cognisant of what constitutes advertising are not inappropriately influenced.* (Food and drink industry responder) (CAP, 2016e)

Arguably, industry responders referred to research evidence to appear knowledgeable and objective, which in turn ‘legitimised’ their involvement within the policy process as it portrays them as credible and informed stakeholders. Furthermore, the majority of industry responders omitted any reference to the obesogenic environment, distancing their framing from highlighting any structural drivers that may reduce the salience of their argumentation.

### 3.4 Recommended policy solutions

Jenkin et al. (2011) propose in their analytical framing matrix that recommended policy solutions should include a consideration of policy prescriptions, non-solutions and existing policies. In general, both industry and non-industry expressed support for the CAP policy proposals. However, the extent of and reasons for this support varied between responder types.

Non-industry responders were supportive of this regulatory improvement as part of a range of policy interventions to reduce the pervasive promotion techniques children were exposed to, often advocating for a blanket ‘ban’ approach to HFSS product advertising and regulatory policy:

*Yes, the placement restriction on HFSS product advertising should be applied to all non-broadcast media, including online advertising, without any exemptions.* (Advocacy organisation responder) (CAP, 2016c)

Non-industry responders often advocated for restrictions that went further than CAP proposed, to ensure a more comprehensive regulatory Code was implemented and to minimise industry’s ability to exploit weak regulatory structures. This was particularly in reference to the proposed rule to allow previously banned advertising techniques to promote non-HFSS products, furthering their framing of the industry as untrustworthy:

*We are very concerned that by allowing any non-HFSS product to be advertised to children using celebrities and licensed characters, there would be many products just under the threshold score for HFSS which would choose to exploit such advertising techniques.* (Government body responder) (CAP, 2016a)

When referring to non-solutions (policy solutions responders considered ineffective), non-industry responders were specific in their rejection of the piecemeal policy proposals, stating they would be unsuccessful at reducing the amount of non-broadcast advertising of HFSS products children were exposed to:

*This guidance does not adopt a sufficiently comprehensive approach. For example, it does not apply to brand equity characters, even though such characters do impact on children’s food preferences*… (Academic responder) (CAP, 2016c)

As such, non-industry responders rejected those regulations that maintained the weak regulatory system, advocating for stringent policy solutions. As can be seen throughout the previous signature rhetorical devices, non-industry responders framed the current CAP policy as ineffective, and rejected the reliance on industry voluntary frameworks:

*It is of [organisation name] view that the current self-regulation for non-broadcast advertising of unhealthy foods and drinks plays an important part in maintaining an obesogenic environment.* (Government body responder) (CAP, 2016d)

On the other hand, industry responders framed themselves as responsible regulators through supporting the proposed policy changes. However, whilst claiming to support the proposals, they continued to insert doubt as to the effectiveness of the changes, by expressing scepticism as to proposals’ ability to reduce childhood overweight and obesity:

*However, obesity and diet are complex issues which will be relatively unaffected by these proposed rule changes.* (Advertising industry responder) (CAP, 2016c)

Whilst supporting the policy proposals as a tool to show industry responsibility, industry responders simultaneously considered the policy proposals as ineffective to resolving the problem of childhood overweight and obesity, framing them as non-solutions. Additionally, they considered some of the proposals as overly-stringent that would negatively impact on their business activities. In particular, industry responders were concerned with the proposal to introduce a 25% audience threshold measure, where HFSS advertising must not be shown where 25% or more of the audience are children:

*[Organisation name] considers that the threshold of 35%, as used in the EU Pledge, is a more appropriate place to find the balance than 25% as proposed by CAP. 25% is disproportionate and is likely to deprive too many adults of the benefits of advertising.* (Food and drink industry responder) (CAP, 2016b)

In contrast to non-industry responders’ call to ban advertising of HFSS products, industry responders advocated for restricting advertising of HFSS products to children, citing existing policy as effective, proportionate, responsible and fair. Industry responders supported the maintenance of the CAP Code, with some improvements, alongside the continued use of their own voluntary frameworks. In some cases, industry responders took this responsibility framing further, by suggesting their organisations’ own voluntary frameworks were more effective than the CAP Code:

*In practice, therefore, much of industry – with advertisers leading the way – is working to (or developing) self-imposed restrictions relating to advertising to children that in some areas are stricter than those found in the CAP Code. It seems sensible to address the apparent disconnect and bring the Code into line with existing good practice.* (Advertising industry responder) (CAP, 2016c)

This was particularly true amongst advertising industry responders, who positioned their industry as an example of best practice in effective policy development. Furthermore, food and beverage industry responders and retailer responders insisted that existing policy provided consumers, and parents in particular, with the information to make healthy dietary choices for their children.

## 4 Discussion

To our knowledge, this study is the first empirical analysis of the public CAP consultation (CAP, 2015) to investigate the framing practices employed by non-industry and industry responders. By conducting a nuanced, detailed analysis of the responses to the CAP consultation, our analysis demonstrates non-industry and industry responders appeared to engage in a moral framing battle centred on whose rights were deemed of most importance to protect – children or industry. The findings further add to previous research demonstrating that consultations are an opportunity for industry and public health advocates to influence regulatory debates, as well as evidence participation within the policy process (Hawkins and Holden, 2013, Razavi et al., 2019, Lauber et al., 2020). Through the examination of both non-industry and industry responses, it was clear that both groups were engaged in a moral framing battle to dominate the political discourse and influence the direction of policy development, by drawing on value frameworks that align with their organisational interests. These frameworks were based on values of fairness and responsibility; noted by Dorfman et al. (2005) as vital to motivating policy change. Building upon these moral frameworks, non-industry and industry responses advocated for similar problem definitions, however varied in their causal interpretations and recommended policy solutions and, again, these aligned with their organisational interests (Petticrew et al., 2017).

Non-industry responders’ overarching moral framework was one based on the protection of children’s rights from exposure to a harmful commodity, aligning with social justice principles of a fair and equal society that protects its most vulnerable (Beauchamp, 1976). As a result, childhood obesity was defined as a public responsibility with the non-broadcast advertising of HFSS products cited as a contributing causal factor to an obesogenic environment (Swinburn et al., 1999). Children were identified as a vulnerable group in need of societal protection from the harmful effects of non-broadcast advertising of HFSS products, and this vulnerability has been highlighted as particularly problematic in the online environment (Kennedy et al., 2019). Similar framing has been employed in other public health debates, and one clear example is that surrounding the implementation of policies to prevent smoking in cars carrying children (Hilton et al., 2014). Through the presentation of children as a group who are particularly vulnerable to the health harms associated with exposure to unhealthy commodities (e.g. HFSS products or cigarettes), public health advocates employed a strategic framing technique to advocate for responsible action to be taken to ensure the health of future generations (Dorfman et al., 2005). This raises the salience of the argument to improve regulatory structures, as it elevates the argument past regulation simply being about limiting industry power to influence dietary behaviours to one that focuses on the end goal of healthy public policy: to ensure children’s ability to attain a healthy diet free from corporate influence.

In contrast, industry responders’ overarching moral framework was one based on the protection of industry rights from perceived disproportionate regulation. Industry responders defined childhood obesity as a problem, aligning with non-industry responders, but minimised the magnitude of the issue. By framing industry as having rights, like the right to advertise, it may be considered that industry organisations in the CAP consultation were using similar arguments employed by industry organisations in the United States (US), where the US Supreme Court ruled in favour of industry as possessing rights similar to human rights. In these rulings, corporations were categorised as ‘people’, and as such possessed similar rights (Hartmann, 2010, Clements, 2012), in a form of ‘corporate personhood’ (Shever, 2010). In this consultation, industry and media organisations repeatedly referred to ‘disproportionate’ regulation that may infringe on their industry rights, framing themselves as victims and their position as one that is vulnerable. As argued by Freudenberg (2014, p134), *“by painting themselves as the beleaguered victims of misguided policies that jeopardize economic growth, corporations and their allies hope to win sympathy from policymakers and the public”*. Again, as seen in previous public health debates involving children as a vulnerable group, industry responders did not attempt to argue that children were not vulnerable but rather highlighted the victimisation that their industry was subject to (Hilton et al., 2014). As such, industry responders recommended policy solutions that aligns with Otero’s (2018) concept of neo-regulation, whereby industry does not simply aim to deregulate but rather to maintain control over the policy process to ensure its alignment with their organisational interests.

In addition to highlighting their industry as victims of perceived disproportionate regulation, industry responders purported to be ‘responsible’ advertisers, and they argued this was demonstrated through their robust self-regulatory system. However, previous evidence suggests that self-regulation serves as a form of CSR initiative (Dorfman et al., 2012), allowing for industry to promote the idea of ‘voluntarism’ (Yoon and Lam, 2013). This process aims to pre-emptively protect industry from perceived risk of disproportionate regulation, and allows them to circumvent unwanted regulation. It also allows industry to simultaneously maintain their rule-setting role, and this has been seen across multiple unhealthy commodity debates (Fuchs and Lederer, 2007). As in previous analyses of self-regulation of UK alcohol advertising, these self-regulatory measures are often employed as part of UCIs’ CSR initiatives to limit government regulation through industry’s emphasis that they are already regulating advertising effectively (Hastings et al., 2010, Hastings and Sheron, 2013). As Yoon and Lam (2013) state, the ‘rosy’ view that industry are better suited to conduct and monitor the rule-setting and rule-following process is problematic, particularly as their profit-generating goals are often at odds with public health goals of improving population health.

Furthermore, industry responders’ emphasis on their responsibility and effective self-regulatory code allows them to point to the ‘real’ problematic actors who were failing in their individual responsibility – consumers and parents. By identifying those who they deemed to be individually responsible, industry responders narrowed who can be blamed for the impact of non-broadcast HFSS advertising, and omitted their own role. Although such findings have been reported as separate CSR initiatives previously (Yoon and Lam, 2013), this study demonstrates that industry responders to the CAP consultation aimed to simultaneously frame themselves as responsible and legitimate policy actors, whilst shifting that responsibility onto consumers and the public. It contributes to an ‘illusion of righteousness’ (Yoon and Lam, 2013), whereby industry overtly portray themselves as morally good and protectors of the vulnerable yet covertly undermine attempts to implement any regulations that would provide that adequate protection. This is an important finding, and as far as the researchers are aware, this is the first it has been evidenced in relation to the industry members associated with the non-broadcast advertising of HFSS products.

Additionally, industry responses appeared to contain several logical fallacies (Petticrew et al., 2017), that make their position difficult to defend. One key fallacy that underpinned industry responses was that they acknowledged that children (albeit aged 11 years and younger) were vulnerable to non-broadcast advertising of HFSS products and required protection whilst simultaneously opposing regulations designed to conduct that protection. In order to defend an arguably morally questionable position, industry responses sought to elevate their rights and consumers’ rights, and advocated for a consideration of those rights rather than argue against the protection of children. A further fallacy was that industry responses tended to state that the problem of childhood obesity was incredibly complex, and therefore we cannot be sure that one single aspect (in this case non-broadcast advertising), is a causal factor. However, these responders pointed to other single factors, for example lack of physical activity, as being a causal factor (yet these were not under consultation for further regulation at the time). Some industry responders referred generally to the scientific evidence base as a means to deflect attention away from population level policies, appearing to conduct a form of ‘evidential landscaping’ whereby they promoted selected evidence of individual-level interventions to oppose population-level policies (Ulucanlar et al., 2014). However, it was not possible to do a full analysis of the evidence used in individual responses as the CAP had removed the reference lists from the responses. These fallacies arguably, as seen in other public health policy debates, seek to infuse doubt into the causes of a public health problem to minimise support for addressing those problems. As noted in Petticrew et al.’s (2017, p1081) study on the use of complexity arguments by food, beverage, alcohol and gambling industries, industry tend to focus on these complexities to argue that *“nothing can be done until everything is done”*.

Furthermore, it is important to comment on non-industry and industry responders’ keenness to express their support for addressing childhood obesity and support for the policy proposals, at least in principle. Drawing on Dorfman et al.’s (2012) concept of industry positioning themselves as ‘good’ corporate citizens through their corporate social responsibility campaigns, it could be argued that in this consultation non-industry and industry responders were positioning themselves as ‘good’ policy citizens. Additionally, by employing such strategic discursive practices, it also serves to position non-industry and industry responders as legitimate policy actors who are willing to engage with and partake in policy debates (Fuchs and Lederer, 2007), in order to avoid criticism that they are ‘not doing their part’.

### 4.1 Limitations

One limitation of this study is that it only included consultation responses, and did not engage with the stakeholders, who contributed to the consultation. This limits the authors’ ability to probe past the written responses. However, the consultation responses represent a ‘snapshot’ of framing practices and this is particularly useful for understanding how key stakeholders respond to proposed regulations, adding to an evidence base which may allow for examinations of frames over time. Due to the nature of the consultation as one in which responders self-selected to contribute, as a data source it cannot be said to provide a representative sample of all non-industry and industry actors. However, those that did respond represent key non-industry and industry actors in the UK policy debate regarding childhood overweight and obesity. The documented frames align with and add to other frames document in wider unhealthy commodity debates suggesting somewhat consistent framing by industry (Yoon and Lam, 2013, Freudenberg, 2014, Petticrew et al., 2017, Lauber et al., 2020). Additionally, this study is one of the first in the UK to consider non-industry actors’ framing in the HFSS product advertising debate.

### 4.2 Policy implications

These findings are timely given the UK Government’s announcement of their programme to address obesity across the UK (UK Government, 2020b), particularly the announcement of increased restrictions on both broadcast and non-broadcast advertising of HFSS products. The findings in this paper demonstrate that industry actors seek to undermine robust public health arguments supporting increased advertising restrictions through the expansion of who is considered a ‘victim’, as well as infuse doubt into the effectiveness of such policies. Additionally, by accounting for the political discourses of all actors within a debate, and the values which underpin these, public health are able to better advocate for strong policy solutions by ensuring that their responses account for and argue against industry logical fallacies that seek to undermine policy recommendations. Non-industry bodies, particularly those within the advocacy sector, can use the findings presented in this research in future consultation responses or broader policy debates to guard against documented industry frames which seek to limit public health policy in a form of counter-framing (Anderson, 2018). A counter-frame is a frame that contradicts the original frame and is introduced at a later date from the original frame. By producing counter-frames that respond to industry’s original frames, rather than generating frames ‘in isolation’, public health advocates may better respond to common industry narratives which seek to limit public health initiatives. Analysing the impact of counter-framing in unhealthy commodity policy debates may also prove a fruitful avenue for future research.

## 5 Conclusion

In conclusion, the analysis of the CAP consultation reports on important findings that non-industry and industry responders were engaged in a moral framing battle that centred on whose rights were deemed worthy of most protection, and drew on principles of fairness, responsibility and equality. This study’s findings are highly relevant to current policy debates on the regulation of both broadcast and non-broadcast advertising of HFSS products to children, as they demonstrate that advocating for policy change, at least in the case of non-broadcast advertising, is structured by key moral frameworks that align with organisations’ interests. This is particularly important in the case of industry responses, as despite emphasising their role as a responsible industry that seeks to protect children, they continue to promote individual responsibility arguments. In addition, industry responses to the consultation, and the self-regulatory code itself, could be seen as a form of CSR initiative, designed to promote industry as a legitimate policy actor and diminish the likelihood of perceived disproportionate regulation that further promotes them as morally ‘righteous’.

By documenting both non-industry and industry frames, the study contributes to a greater understanding of the importance of moral frameworks in the construction of arguments to advocate for or against suggested policy solutions to non-broadcast HFSS product advertising regulation (Dorfman et al., 2005). This may assist public health advocates in their campaigning strategies, as it allows for such advocates to potentially produce counter-frames to the industry frames explored above. This would strengthen their public health advocacy goals, as well as potentially limit the effectiveness of industry framing in undermining the strengthening of regulation of non-broadcast HFSS advertising.

## Funding

LCW was supported by a Medical Research Council PhD studentship (MC_ST_U15006), a MRC Transition Fellowship (MR/N0131661/1) and the UK Research and Innovation Councils SPECTRUM Consortium (MR/S037519/1). SC was supported by a Medical Research Council Strategic Award (MC_PC_13027), Medical Research Council grants (MC_UU_12017/14; MC_UU_12017/12; MC_UU_00022/1) and Chief Scientist Office of the Scottish Government Health Directorates grants (SPHSU12; SPHSU14; SPHSU16). SH was supported by Medical Research Council grant (MC_UU_12017/15) and the Chief Scientist Office of the Scottish Government Health Directorates Grant (SPHSU15). KS was supported by a Medical Research Council Strategic Award (MC_PC_13027), Medical Research Council Grants (MC_UU_12017/11; MC_UU_00022/3), and the Chief Scientist Office of the Scottish Government Health Directorates grants (SPHSU11; SPHSU18).

LCW is a member of SPECTRUM a UK Prevention Research Partnership Consortium. UKPRP is an initiative funded by the UK Research and Innovation Councils, the Department of Health and Social Care (England) and the UK devolved administrations, and leading health research charities.

## CRediT authorship contribution statement

**Lauren Carters-White:** Conceptualization, Methodology, Data curation, Resources, Formal analysis, Writing - original draft, Writing - review & editing, Funding acquisition. **Stephanie Chambers:** Conceptualization, Methodology, Supervision, Writing - review & editing, Funding acquisition. **Kathryn Skivington:** Conceptualization, Methodology, Supervision, Writing - review & editing, Funding acquisition. **Shona Hilton:** Conceptualization, Methodology, Supervision, Writing - review & editing, Funding acquisition.

## Declaration of Competing Interest

The authors declare that they have no known competing financial interests or personal relationships that could have appeared to influence the work reported in this paper.
